# How to activate students’ natural desire to test themselves

**DOI:** 10.1186/s41235-019-0187-y

**Published:** 2019-09-23

**Authors:** Kalif E. Vaughn, Nate Kornell

**Affiliations:** 1 Northern Kentucky University, Highland Heights, KY USA; 2 Department of Psychology, Williams College, Williamstown, MA USA

**Keywords:** Self-testing, Hints, Memory

## Abstract

Testing oneself (i.e., doing retrieval practice) is an effective way to study. We attempted to make learners choose to test themselves more often. In Experiment 1, participants were asked how they wanted to study and were given four options: retrieval with no hint (e.g., idea: ______), a two-letter hint (e.g., idea: s____r), a four-letter hint (e.g., idea: se__er), or a presentation trial (e.g., idea: seeker). They tested themselves on the majority of trials. In Experiment 2, when the hint options were removed, they chose restudy rather than pure test on the majority of trials. These findings show that people prefer self-testing over restudy as long as they can get the answer right on the test. However, we would not recommend hints if they impaired learning compared to pure testing. Experiment 3 showed that this was not the case; the three retrieval conditions from Experiment 1 led to equivalent amounts of learning, and all three outperformed the pure presentation condition. We used different materials in Experiment 4 and found that the hints made retrieval slightly less beneficial when the hints made it possible to guess the answers without thinking back to the study phase (e.g., whip: pu__sh). In summary, hints catalyzed people’s intuitive desire to self-test, without any downside for learning, thus making their self-regulated study more enjoyable and effective.

## Significance

Self-testing represents a beneficial learning strategy, and we sought to make students want to self-test more often. We utilized hints to make self-testing more desirable. In Experiment 1, participants were able to choose from a pure test trial (idea: ______), a two-letter hint (idea: s_____r), a four-letter hint (idea: se__er), or a pure study trial (idea: seeker). They tested themselves on the majority of the trials, but this preference was reversed in Experiment 2 when we removed the hint options. Experiment 3 demonstrates that these hints do not decrease learning insofar as the target is not guessable. When the target is guessable, the hints make the test trials less effective (Experiment 4). Overall, this research demonstrates that learners prefer self-testing when the tests are made more palatable with hints. Furthermore, these hints do not decrease learning if the target is unguessable. Without hints, students avoid self-testing; with hints, students are more likely to self-test, find self-testing more enjoyable, and learn just as much as testing without hints.

## How to activate students’ natural desire to test themselves

Can you name the eight US states whose names begin with the letter N?^1^ How about the eight that start with M?^2^ Do you know the name of Batman’s butler?^3^ Draco Malfoy’s aunt?^4^ Thor’s hammer?^5^ Nirvana’s drummer?^6^ The family in The Incredibles, The Godfather, or The Sound of Music?^7^ And what musical groups popularized the song Colors, Black, Behind These Hazel Eyes, Blue Suede Shoes, Brown Eyed Girl, Purple Rain, Mellow Yellow, Green Onions, Pink Moon, Black Dog, Blackbird, Back in Black, Back to Black, Paint it Black, Fade to Black, Black Hole Sun, and White Room?^8^

Answering questions can be enjoyable. If these questions did not tickle you right, then others surely would. We suspect that some readers lingered on that first paragraph, trying to think of the answers. Checking the footnote would have been faster, and if you have a lot to do it might have been the rational or mature thing to do, but it would also spoil the fun. Part of the fun comes from the content of the question being interesting, but interestingness cannot be the whole story because the content of the question does not change depending on whether you think of the answer or it is told to you. Part of the fun is surely meeting the challenge of a question by coming up with the answer. It is more rewarding to retrieve the answer to a question than to have it told to you or look it up.

Answering questions is not just fun, it is also a good way to learn. People learn more when they retrieve information from memory than when they are told the same information (Roediger & Butler, 2011; Rowland, 2014). Retrieval practice boosts memory for foreign language word pairs (Fritz, Morris, Acton, Voelkel, & Etkind, 2007), can improve learning in schools (Roediger, Agarwal, McDaniel, & McDermott, 2011), and has even been shown to produce more learning than elaborative study strategies such as concept mapping (Karpicke & Blunt, 2011).

However, retrieval is only beneficial if you actually do it. Unfortunately, there is some evidence that students take a dim view of testing. Surveys show that many students report that they do not test themselves as they study and experimental studies show that they rate restudy as more effective than self-testing (Geller et al., 2018; Hartwig & Dunlosky, 2012; Karpicke, Butler, & Roediger, 2009; Karpicke & Roediger, 2008; Kornell & Bjork, 2007; Kornell & Son, 2009; Morehead, Rhodes, & DeLozier, 2016; Wissman, Rawson, & Pyc, 2012). If students choose restudy over testing, they may be hurting their own learning.

Indeed, there is empirical evidence that learners avoid self-testing when given the choice. Karpicke (2009) had participants study and recall items once. Once an item was recalled, learners could either restudy the item, take a test on the item, or drop the item from further practice. Results showed that participants dropped the majority of items from practice after one correct recall. This is not the optimal strategy. Vaughn and Rawson (2011) showed that repeatedly retrieving items during practice improves memory for cues, targets, and their associations. Many others have shown the benefits of repeated retrieval practice (e.g., Karpicke & Roediger, 2007; Pyc & Rawson, 2011; Vaughn, Rawson, & Pyc, 2013). Clearly, memory is best when learners continue to self-test beyond one correct recall. The problem is motivating learners to want to self-test.

However, things might not be as bad as they seem. For one thing, what matters is how students choose to study, not what they think is best for learning. Choices and beliefs are not the same. For example, in a study by Kornell and Son (2009), most participants indicated that restudying was the most effective way to study but, when asked how they wanted to study, the majority chose to test themselves. This is inconsistent with the findings reported by Karpicke (2009). Methodological differences aside, it turns out that learning efficiency was not the only goal of these participants; when asked why they chose to test themselves, participants often said it helped them find out how well they knew the material. Thus, tests are appealing for self-monitoring, a reason that is not directly about learning. We think there is a second reason tests are also appealing: given the right conditions, we think students enjoy being tested.

### Do learners like to take tests?

We began with the hypothesis that people like to be tested as long as they have a good chance of getting the answer right. When people avoid tests, and choose restudy instead, we hypothesize that they are not trying to avoid taking a test per se, but that they are trying to avoid failure; they do not want to get the answer wrong. Thus, when a person does not have good knowledge of the information they prefer restudy, but when they reach a certain level of competence they prefer to be tested. We do not wish to suggest that learners will only test themselves if they can get the answer correct. Rather, we think learners will self-test at higher rates as they think their odds of being correct increase (i.e., as stronger versus weaker hints become available). Moreover, we think that, overall, they would rather be taking tests than restudying; that is, they would rather face a set of test questions they might be able to get right than a set of presentation trials on items they do not know well. We attempted to test these hypotheses in the present research.

In support of these hypotheses, Kornell and Bjork (2007) allowed participants to choose how they wanted to study the same set of word pairs across multiple blocks of trials. The first time working through the pairs, participants almost never chose to test themselves, instead choosing to see the cue and target together. As they gained more experience with the pairs, however, they switched to testing themselves (i.e., seeing the cue and trying to think of the target, then checking the target) the majority of the time. In short, they preferred testing if they could get the answer right. For similar results, see Tullis, Finley, and Benjamin (2013).

Additional support for our hypotheses comes from our intuition that, as stated earlier, people like tests. This is why “wait, wait, don’t tell me!” is both an idiom and a popular radio show; answering questions is more enjoyable than being told the answers. Anecdotal evidence also suggests that most people intuitively believe testing is a good way to learn. For example, in Quizlet, the wildly popular learning app that had over 50 million active users per month as of October 2018 (Clark, 2018), students can choose to study using a variety of modes (e.g., flashcards, learn, write, spell, test), but all of these modes involve retrieval. Students who use the app are implicitly buying in to the idea that testing is the most effective way (and the only way, in the app) to learn the material they learn on Quizlet. The intuition that testing is the appropriate way to learn is so strong that we have caught participants in laboratory experiments covering the target word in a word pair with their hand so they could test themselves, effectively turning a restudy condition into a test condition. This ruined our control condition, but it also testifies to the strong impulse students have to test themselves on material they know well.

### The present experiments

Our ultimate goal was to increase learning by getting participants to test themselves more. However, we did not want to create a situation where our participants would have preferred to choose restudy but chose testing anyway. We wanted our participants to want to test themselves while they studied. To do this, we let them decide the difficulty of the test trials.

In Experiment 1, participants could choose pure study trials or pure test trials, but they also had the option to take tests with hints, allowing them to choose a trial type that allowed them to be tested but also get the answer correct. In Experiment 2 the hint options were removed. We predicted that they would try to test themselves as long as they could avoid getting answers wrong. Thus, we predicted that participants would choose test-with-hint trials in Experiment 1, because these trials would allow testing themselves without getting the answers wrong. We predicted that they would choose presentation trials in Experiment 2, when pure tests were the only alternative, because they were likely to get answers wrong if they chose pure test trials. Thus, Experiment 1 and 2 were designed to evaluate self-testing preferences when hints were available (Experiment 1) versus not available (Experiment 2).

Experiment 3 and 4 were designed to examine learning. Our ultimate goal was to improve learning. If hints encourage people to test themselves, that is a good first step. But what if the tests with hints are no more effective than presentations? Then we would not recommend hints as a way to help students learn more efficiently. Hints would only be truly beneficial if they do not destroy the benefits of retrieval. Thus, we examined how much participants learned from pure test trials, hint trials, and pure study trials. In Experiment 3a, we used unrelated word pairs (e.g., idea-seeker) to examine the effects of these hints when the word pairs were not guessable. Experiment 3b was a replication of Experiment 3a with a different population. In Experiment 4, we changed the materials from unrelated word pairs to weakly related word pairs (e.g., whip-punish) to examine the effect of hints when the word pairs were guessable.

## Experiment 1

In Experiment 1, participants decided how to study each item during the study phase. On each trial they were shown a cue word (e.g., idea). They controlled how many letters of the target (0, 2, 4, or all 6) were shown. They could choose to see zero letters (e.g., idea-______), a two-letter condition (e.g., idea-s____r), a four-letter condition (e.g., idea-se__er), or all six letters (e.g., idea-seeker). The first of these trial types is a pure test, the next two are tests with hints, and the last is a pure presentation. We predicted that participants would take advantage of the hints to ensure they would have a good chance of getting the answers correct during the study phase, and in doing so would prefer some sort of test trial over pure presentation. In other words, we offered participants hints so that they would engage in retrieval practice.

### Method

#### Participants

We calculated power by assuming a medium size effect (η^2^_p_ = .06) based on previous studies that have examined preferences for testing versus restudy. We needed 30 participants to achieve a power = .90, with alpha = .05. We requested more than this to account for participants who did not finish the study as requested.

Fifty-one participants started the experiment. Of these, 10 restarted part-way through and three people did not finish. These participants were excluded from subsequent analyses. Of the remaining 38 participants, one participant indicated that he should be excluded from the analyses because there were major problems during the study. Additionally, we excluded one person who failed to correctly copy the target words during the study phase (mean copy performance was only 1.7%).

In the end, 36 participants (22 female, 14 male; median age 32.5 years, range 22–61 years) completed the experiment on Amazon’s Mechanical Turk. Participants were compensated $5.00 for completing the experiment. All participants reported being fluent English speakers living in the United States.

#### Materials

Sixty unrelated word pairs were constructed such that: 1) the cues and targets were unrelated; 2) all target words were six letters long; 3) the target words occurred with moderate frequency in the English language; and 4) all test trial hints (e.g., idea: se__er) allowed for at least five possible alternatives answers (e.g., sexier, server, sender, seller, seeker). This last constraint was important because it insured that, when the hint was shown, participants had to rely on their episodic memory of the prior study trial to come up with the answer. If there had only been one possible word consistent with the hint, participants could have relied on semantic memory to guess the target correctly simply by looking at the hint.

To construct this word set, we first developed a list of words divided into sets such that all words in a set had the same letter as the others in the first, second, fifth, and sixth position. We then excluded any word that had a CDcount below 25 in the Subtlexus database (e.g., Brysbaert & New, 2009), which insured that all words had at least a moderate frequency as measured by movie subtitle counts. Any set of words that had at least five possible alternatives, given these constraints, was considered for use in our study. We then selected the fifth most frequently used word among each set as the target word. For a list of the five most common words, see Appendix 1 (a table containing every word in each set, along with its CDcount, is available in Appendix 1). We then constructed a set of four-letter nouns to serve as cues. The cues and targets were paired such that their forward and backward association strength were both zero (Nelson, McEvoy, & Schreiber, 2004). Example word pairs include “love: banter”, “gene: bonded”, and “road: cordon” (see Appendix 1 for a full list of word pairs).

#### Procedure

The experiment was conducted online. The word order was randomized in each phase of the experiment for each participant. During the initial phase of the experiment, all sixty word pairs were shown in copy trials. During a copy trial, the cue and target were both presented on the screen (e.g., idea: seeker), and the participant was instructed to type the target word into the textbox. To ensure that participants looked at both words in the pair before they began typing, there was a delay of 2 s between the time when the words appeared and the time when the textbox and submit button appeared.

After the initial study phase, the restudy phase commenced. On each trial, before a word pair was shown, participants were asked how they would like to study the next pair. They were shown four response options: “0 Letters (e.g., wolf - ______)”, “2 Letters (e.g., wolf - k____t)”, “4 Letters (e.g., wolf - kn__ht)”, and “6 Letters (e.g., wolf - knight)”. Participants made this choice prior to seeing the cue word. This was done intentionally; if participants saw the cue word before making their choice, they would likely attempt retrieval. This would render each trial a “0 Letters (e.g., wolf - ______)” followed by a subsequent trial based on their choice. We wanted participants to make their choice based only on their learning preferences, not after they had given themselves an initial test on the item. Once they chose an option, they were shown the cue and target in accordance with their choice. The pair was shown for 4 s before the textbox for answering and submit button appeared. This delay was used to ensure that participants processed the cue and attempted retrieval for at least 4 s and that all the test trial conditions received a similar amount of exposure during practice. At the end of each trial, after the participant had submitted their response, they received feedback. During a feedback trial, the cue and target were both presented on the screen (e.g., idea: seeker). After 2 s of displaying the cue and target on the screen, the submit button appeared and participants could advance to the next trial. The cue and target remained on the screen until the submit button was pressed. This process repeated until all 60 items had been tested.

At the end of the study, participants were asked three final questions about their experience with the four conditions they had experienced. These questions are displayed in Table 1. They were also asked whether they experienced any major problems during the study (e.g., internet connectivity issues, page loading issues, and so on) and whether they were distracted during the experiment and/or to list the other activities they were working on during the task. Participants did not report any major problems or distractions during the experiment. On average, participants completed the experiment from start to finish in 36 min 8 s.

**Table 1:** Percentage of choices on the final questionnaire for each trial type in Experiments 1 and 2

	Number of letters			
	Zero	Two	Four	Six
Which trial do you feel best helped you learn the word pairs?				
Experiment 1	0.0	5.7	54.3	40.0
Experiment 2	12.8	–	–	87.2
If you could only use one trial type from now on, which would you choose?				
Experiment 1	0.0	5.7	54.3	40.0
Experiment 2	15.8	–	–	84.2
Which trial type was the most fun?				
Experiment 1	0.0	17.1	71.4	11.4
Experiment 2	41.0	–	–	59.0

### Results

**Fig. 1:**
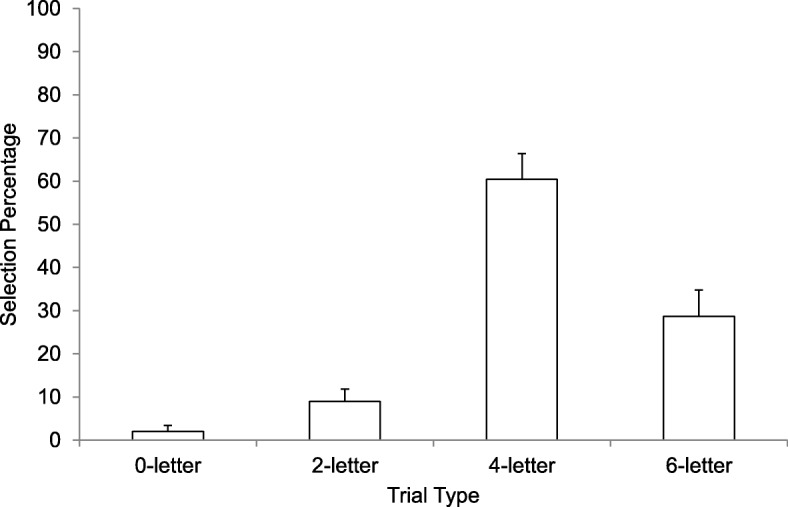
Selection rate (reported as a percentage) as a function of trial type in Experiment 1. Error bars report standard error of the means

Items not correctly copied on the initial copy trial were discarded from subsequent analyses (23/2152 trials; 1.07%). Figure 1 shows how often participants selected each trial type during the initial test phase. A Greenhouse-Geisser repeated-measures analysis of variance (ANOVA) revealed a significant main effect of trial type choice during practice, *F* (1.46, 51.13) = 24.79 *p* < .001, η^2^_p_ = .42. Holm post-hoc comparisons revealed significant differences in selection rates between all trial types (all *p* values < .029).

Answers to the final questions are reported in Table 1. Overall, the four-letter trial type was endorsed most often as the most effective, the most fun, and as the trial type participants would prefer to use from now on.

There are two important points to be made regarding these final questions. First, no-one selected the zero-letter trial for any of the questions. This shows how averse participants are to testing without hints. Second, although testing with hints was the preferred strategy, the preference was not overwhelming given that approximately 40% of the participants still chose the six-letter trial (restudy) option as the one that helped them learn the most and the one they would use from now on.

In summary, our participants chose to test themselves on the majority of trials. Our results were consistent with past studies in the sense that participants chose pure restudy far more often (29% of trials) than pure test (2% of trials) (e.g., Karpicke, 2009; Kornell & Bjork, 2007). However, the addition of hint trials appears to have given participants a more preferred option. They chose two-letter hints and four-letter hints on 9% and 60% of trials, respectively, resulting in a test trial of some kind being chosen 71% of the time. These results are encouraging because students learn more when they test themselves and these results showed that hints are an effective way to get them to test themselves.

## Experiment 2

In Experiment 2 we examined how often participants would test themselves when hints were not available. We were not sure whether the presence of hints actually affected participants’ use of retrieval. The results of Experiment 1 suggested that they would prefer pure restudy to pure testing. However, there is an alternative possibility; on trials where they chose a hint, perhaps they would have chosen test trials if no hints had been available. If so, they might still have tested at the same rate as they did in Experiment 1, around 70% of the time. We hypothesized that they would choose to test themselves less often than that and, indeed, less than 50% of the time.

### Method

#### Participants

We calculated power by assuming a large size effect (*d* = .80) based on Experiment 1. We needed 19 participants to achieve a power = .90, with alpha = .05. We chose more than that to account for participants who did not finish the study as requested.

Fifty-two participants started the experiment; however, six people were excluded from the analyses because they either restarted or did not finish the experiment. One participant was also excluded from the analyses due to indicating that they had previously completed the experiment. As an additional safeguard, we cross-referenced the current list of participants with a master list of participants who had completed other versions of this experiment under a different HIT on mTurk. That comparison revealed that five participants had completed a prior version of this experiment; those participants were excluded from the analyses. Additionally, we excluded one person who failed to correctly copy the target words during the study phase (mean copy performance was 0%).

In summary, 39 participants (14 female, 25 male; median age 32 years, range 21–68 years) completed the experiment on Amazon’s Mechanical Turk. Participants were compensated $5.00 for completing the experiment. All participants reported being fluent English speakers living in the United States.

#### Procedure

The materials and procedure were identical to those used in Experiment 1 except for one key difference. When participants were asked how they would like to study the next pair, they were shown only two response options: “0 Letters (e.g., wolf - ______)” or “6 Letters (e.g., wolf – knight)”. On average, participants completed the experiment from start to finish in 31 min 12 s.

### Results

**Fig. 2:**
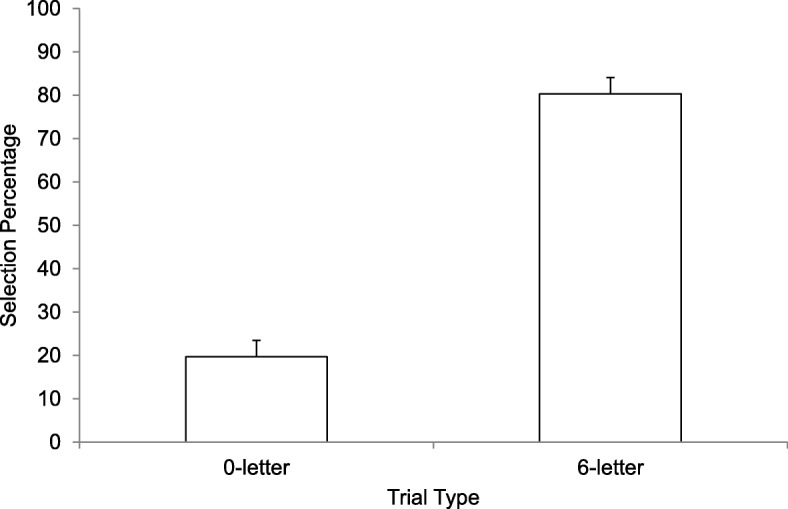
Selection rate (reported as a percentage) as a function of trial type in Experiment 2. Error bars report standard error of the means

Items not correctly copied on the initial copy trial were discarded from subsequent analyses (8/2340 trials; 0.34%). How often participants selected each trial type during the initial test phase is shown in Fig. 2. Participants selected the six-letter condition significantly more often than the zero-letter condition, *t*(38) = 8.08, *p* < .001, *d* = 1.29, 95% confidence interval (CI) 45.5–75.8. Responses to the final questions are reported in Table 1. The six-letter trial type was endorsed as the most effective, the most fun, and the trial type participants would prefer to use from now on. This preference was relatively strong given that 87% thought restudying was more effective than testing without hints and 84% would use restudy from now on. Interestingly, restudying was only seen as slightly more fun than testing without hints.

In summary, participants chose pure restudy on the majority of trials. This finding is consistent with the data from the no-hint trials in Experiment 1 and previous research (e.g., Roediger & Butler, 2011).

Taken together, Experiments 1 and 2 showed that hints made a difference to the decisions of participants about how to study. When hints were available, participants tested themselves about 71% of the time, compared to 20% of the time when hints were not available (in Experiments 1 and 2, respectively). Thus, hints seem to entice people to test themselves while they study.

We did not examine final test performance in Experiment 1 and 2 because the number of items contributing to each condition varied substantially across participants. Moreover, there were two reasons to expect that the condition would be confounded by other factors. First, order effects seemed possible (e.g., a participant might start with presentation trials and then shift to test trials) and, second, participants who found the task easier overall might have done more test trials during the study, and performed better on the final test, than participants who found the task more difficult. Thus, any final test comparisons were potentially confounded, and would have been uninterpretable. Experiments 3 and 4 were able to examine the impact of hints on final test performance because items were assigned to conditions randomly.

## Experiment 3

The next step was to find out what effect the hints had on learning. What effect does a retrieval trial with a hint have on learning? If it is no better than a presentation trial, then encouraging people to do hint trials might not improve their learning. If it is as good as a pure retrieval trial, on the other hand, then hint trials could be very useful for students—they would do more retrieval and the value of that retrieval would be undiminished.

Experiment 3b was a replication of Experiment 3a, so their procedures are the same. Their procedures were also very similar to those of Experiment 1. There were two main differences. First, the participants did not choose how they would study. Items were randomly assigned to one of the four conditions from Experiment 1. Second, there was a final cued-recall test.

## Experiment 3a

### Method

#### Participants

We calculated power by assuming a medium size effect (η^2^_p_ = .06) based on previous studies that have examined the effect of retrieval on learning. We needed 30 participants to achieve a power = .90, with alpha = .05. We chose more than that to account for participants who did not finish the study as requested.

Forty-six participants started the experiment, but one person restarted and three participants did not finish the experiment. These participants were excluded from subsequent analyses. Additionally, one person failed to copy any of the items correctly during practice and was excluded from subsequent analyses.

In summary, 41 participants (17 female, 24 male; median age 32.5 years, range 22–57 years) successfully completed the experiment on Amazon’s Mechanical Turk (we excluded the age of one participant who indicated that he was 2 years old). Participants were compensated $5.00 for completing the experiment. All participants reported being fluent English speakers living in the United States.

#### Procedure

The procedure was the same as for Experiment 1 with two exceptions. First, during the restudy phase, the participants were not asked to decide how they would study. Instead, fifteen pairs were randomly assigned (uniquely for each participant) to each of four conditions: the zero-letter condition (e.g., idea: ______), the two-letter condition (e.g., idea: s____r), the four-letter condition (e.g., idea: se__er), and the six-letter condition (e.g., idea: seeker).

Second, there was a short delay followed by a final test. After the restudy phase ended, participants played the video game ‘Tetris’ for 2 min. After this distractor phase, participants began the final test. The cue word and an empty textbox were presented on each trial (e.g., idea: ______). No feedback was provided during the final test phase. On average, participants completed the experiment from start to finish in 39 min 53 s.

### Results

Items not correctly copied on the initial copy trial were discarded from subsequent analyses (83/2520 trials; 3.3%). Response accuracy was determined using an algorithm that counted slightly misspelled words as correct. This scoring method was used on the initial and final test in Experiments 3a, 3b, and 4.

**Fig. 3:**
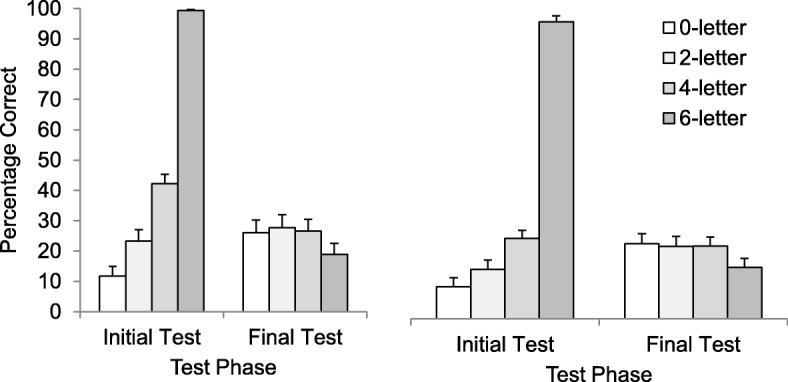
Initial and final recall performance (reported as a percentage) as a function of trial type in Experiment 3a (left panel) and Experiment 3b (right panel). Error bars report standard error of the means

A Greenhouse-Geisser repeated-measures ANOVA revealed a significant effect of condition on initial test performance, *F* (2.27, 90.66) = 344.20, *p* < .001, η^2^_p_ = .90. As shown in Fig. 3, during the restudy phase participants answered correctly more often when more letters of the target were visible.

Final test performance is plotted in Fig. 3 (left panel). We analyzed final test response accuracy in two ways. First, we used a Greenhouse-Geisser repeated-measures ANOVA to compare final test performance across all four conditions. The analysis revealed a significant main effect of condition, *F* (2.39, 95.44) = 6.37, *p* = .001, η^2^_p_ = .14.

Our main question was whether retrieval with hints was less beneficial than retrieval without hints. Therefore, in a second analysis we examined only the three retrieval conditions (i.e., the zero-letter, two-letter, and four-letter conditions). For this analysis, a Greenhouse-Geisser repeated-measures ANOVA revealed no significant difference in final test performance, *F* (1.95, 77.91) = 0.39, *p* = .672, η^2^_p_ = .01.

Additionally, Holm post-hoc pairwise comparisons were made across all four learning conditions. These pairwise comparisons were consistent with the results from the repeated-measures ANOVAs. First, the six-letter condition exhibited significantly worse final test performance than the four-letter condition (*p* = .004, *d* = 0.57), the two-letter condition (*p* = .010, *d* = 0.52), and the zero-letter condition (*p* = .047, *d* = 0.41). There was no difference between the retrieval conditions (all *p* values > .99).

In summary, Experiment 3a showed that more learning occurred in the three retrieval conditions than in the presentation condition. More importantly, there were no significant differences between test trials without hints and test trials with hints. In other words, hints did not appear to impact learning.

## Experiment 3b

We decided to replicate Experiment 3a for two reasons. First, the results do not necessarily fit with prior research or theory. According to the desirable difficulty framework, participants should learn more when learning conditions are made more difficult (Bjork, 1994; Bjork & Bjork, 1992, 2011). In contrast, we found no difference between relatively easy hint trials and more difficult no-hint trials. Furthermore, according to the retrieval effort hypothesis, more difficult retrieval trials should produce more learning than easier retrieval trials (Bjork & Allen, 1970; Pyc & Rawson, 2009). Our data showed that easier and more difficult retrieval trials produced equivalent learning. (We will return to these points in the General discussion.) Second, the main conclusion of Experiment 3a hinged on a null result and we wanted to know whether the findings would hold up with a larger sample drawn from a different population.

### Method

#### Participants

One hundred and thirty participants started the experiment, more than twice as many as started Experiment 3a. Thirty-seven participants did not finish the experiment, four participants indicated that they had completed a prior version of this experiment before, and 21 participants wished to be excluded. These participants were excluded from subsequent analyses. Additionally, five participants failed to copy a significant portion of the items correctly during the copy trials (mean copy performance 26.67%) and were excluded from the subsequent analyses.

In the end, 63 participants (45 female, 18 male; median age 19 years, range 17–59 years) successfully completed the experiment online at Northern Kentucky University. They were compensated with course credit. All participants reported being fluent English speakers (except for two participants who provided a nonsensical answer of ‘0’, which is presumably the result of computer error). All participants indicated that they lived in the United States, except for one participant who had a ‘0’ for this answer, one participant who answered ‘Nepal’, and another who answered ‘South Africa’. Given that this experiment was only advertised to Northern Kentucky University students, it is likely that these students originated from these other countries but currently resided in the United States (indeed, one indicated that she lived in ‘Ohio’ on a separate question). Therefore, these participants were not excluded from the analyses.

The materials and procedure were identical to those used in Experiment 3a. On average, participants completed the experiment from start to finish in 34 min 57 s.

### Results

Items not correctly copied on the initial copy trial were discarded from subsequent analyses (11/3775 trials; 0.29%). A Greenhouse-Geisser repeated-measures ANOVA was used to analyze initial test performance. Replicating Experiment 1a, providing more letters made initial retrieval more successful, *F* (2.01, 124.81) = 449.24, *p* < .001, η^2^_p_ = .88 (see Fig. 3).

Final test performance is plotted in Fig. 3 (right panel). We conducted two ANOVAs, and both replicated the findings of Experiment 1a. When all four conditions were compared, a Greenhouse-Geisser repeated-measures ANOVA revealed a significant effect of condition, *F* (2.59, 160.84) = 8.01, *p* < .001, η^2^_p_ = .11. When we analyzed only the three retrieval conditions (i.e., the zero-letter, two-letter, and four-letter conditions), a Greenhouse-Geisser repeated-measures ANOVA revealed no significant effect of condition, *F* (1.77, 109.53) = 0.11, *p* = .874, η^2^_p_ = .002.

Additionally, Holm post-hoc pairwise comparisons were made across all four learning conditions. The six-letter condition exhibited significantly worse final test performance than the four-letter condition (*p* < .001, *d* = 0.61), the two-letter condition (*p* = .003, *d* = 0.45), and the zero-letter condition (*p* < .001, *d* = 0.56). There was no difference between the retrieval conditions (all *p* values > .99).

In summary, Experiment 3b replicated the results of Experiment 3a. The three retrieval conditions produced the same amount of learning, regardless of whether participants were given hints. All three conditions were more effective than the presentation condition. These findings suggest that if students were given the option to test themselves with hints, they might test themselves more without suffering any detriment to the amount they learned from the test trials.

## Experiment 4

In the first three experiments, participants learned the same set of word pairs—unrelated cue-target pairs that were especially chosen so that the target was not guessable even with a four-letter hint. It took a great deal of careful effort to come up with these pairs, and they were considered high maintenance. We wanted to see whether the results of Experiment 3 would generalize to a lower-maintenance set of materials because if these effects only happen with high-maintenance materials there would need to be limitations on any recommendations we might make about how and when to give hints. Therefore, the materials in Experiment 4 were related word pairs (e.g., whip-punish; see Appendix 2 for a full list of the related cue-target pairs used in Experiment 4).

### Method

#### Participants

We calculated power by assuming a large size effect (η^2^_p_ = .10) based on Experiments 3a and 3b. We needed 18 participants to achieve a power = .90, with alpha = .05. We chose more than that to account for participants who did not finish the study as requested.

Fifty-three participants started the experiment; however, fourteen were excluded because they either restarted or did not finish. One participant was excluded because she indicated that she had completed the experiment before. Additionally, we excluded two people who failed to correctly copy many of the target words during the study phase (mean copy performance was 31.7%). In summary, 36 participants (23 female, 12 male, 1 person did not enter their gender; median age 35 years, range 21–69 years) completed the experiment on Amazon’s Mechanical Turk. Participants were compensated $5.00 for completing the experiment. All participants reported being fluent English speakers living in the United States.

#### Materials and procedure

The materials were 60 weakly related cue-target pairs (e.g., whip: punish). The average forward associative strength of the word pairs was .053 (Nelson et al., 2004). The procedure was identical to the procedure used in Experiments 3a and 3b. On average, participants completed the experiment from start to finish in 32 min 43 s.

### Results

**Fig. 4:**
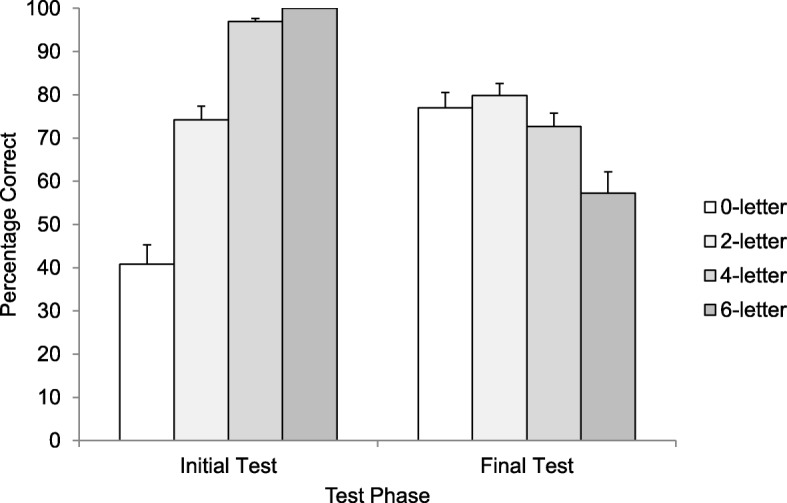
Initial and final recall performance (reported as a percentage) as a function of trial type in Experiment 4. Error bars report standard error of the means

Items not correctly copied on the initial copy trial were discarded from subsequent analyses (3/2160 trials; 0.14%). A Greenhouse-Geisser repeated-measures ANOVA revealed that initial test performance increased as more letters of the target word were provided, *F* (1.39, 48.74) = 140.10, *p* < .001, η^2^_p_ = .80 (see Fig. 4).

Final test performance as a function of initial trial type is plotted in Fig. 4. When comparing final test performance across all four conditions, a Greenhouse-Geisser repeated-measures ANOVA revealed a significant main effect, *F* (2.58, 90.44) = 25.17, *p* < .001, η^2^_p_ = .42. We also compared only the three retrieval trial types (i.e., zero-letter, two-letter, and four-letter conditions) using a Greenhouse-Geisser repeated-measures ANOVA. In contrast to Experiments 3a and 3b, there was a significant difference between conditions, *F* (1.71, 59.78) = 4.04, *p* = .028, η^2^_p_ = .10.

Post-hoc Holm comparisons suggested that all retrieval trial types (i.e., zero-letter, two-letter, and four-letter conditions) yielded significantly better recall performance compared to the restudy trial type (i.e., six-letter condition) (all *p* values < .001). There was also a significant difference between the two-letter and four-letter conditions (*p* = .009, *d* = 0.54). The zero-letter condition did not differ significantly from the two-letter condition (*p* = .312) or the four-letter condition (*p* = .312).

Why did the retrieval conditions differ here but not in Experiment 3a or 3b? We suspect the answer has to do with guessability. In Experiments 3a and 3b, the cue-target word pairs were nonguessable regardless of whether we provided zero letters (e.g., idea: ______), two letters (e.g., idea: s_____r), or four letters (e.g., idea: se__er). In Experiment 4, the cue-target word pairs were all weakly related. Thus, the targets are likely guessable when we provided letter hints, especially when we provided four letters (e.g., whip: pu__sh). If participants could guess the target words, then they did not actually have to try to retrieve a memory of their prior encounter with the pair (from the study phase). In other words, engaging in episodic retrieval was the only way to think of the target in the restudy phase of Experiments 3a and 3b, but in Experiment 4 episodic retrieval was not necessarily required because the targets became guessable.

In summary, Experiment 4 replicated Experiments 3a and 3b in the sense that the retrieval conditions were all more effective than presentation. The results also differed, however, in that the amount participants learned from retrieval was affected by hints. Specifically, there was a significant difference between the two-letter versus four-letter retrieval conditions. We speculate that this difference came about because the four-letter hint made it possible for participants to guess the target without engaging in episodic retrieval. Furthermore, unlike the prior experiments, we did not ensure that multiple answers were possible when participants were provided with the hints.

## General Discussion

To summarize, when our participants were offered two options, either restudy or taking a test, they chose restudying on the majority of trials. When they were allowed to request hints during test trials, however, they preferred testing over restudy by a sizeable margin. In other words, difficult test trials were like black coffee (revolting) while test trials with hints were like coffee with milk and sweetener (heavenly). In terms of learning, we found that participants learned more from any kind of test trials than they did from restudy trials, but learning was not affected by hints.

These results suggest that making retrieval easier by giving hints might be an effective way to increase learning. The point was not to change the learning efficiency of retrieval or restudy trials, it was to make people prefer to study in a more efficient way. Retrieval is considered a desirable difficulty because it makes learning more difficult in the short term but enhances learning in the long term (e.g., Bjork, 1994; Bjork & Bjork, 2011). Unfortunately, desirable difficulties are not always desirable to the learner because learners typically, but incorrectly, assume that poor short-term performance is equivalent to poor learning (for reviews, see Bjork, Dunlosky, & Kornell, 2013; Soderstrom & Bjork, 2015). Retrieval with hints appears to be a rare case of ‘desirable easiness’; it was similar to a desirable difficulty in terms of the long-term benefits of retrieval being desirable for learning, but it was different in the short term because the easiness brought on by the hints made learners find retrieval desirable as well.

### Practical recommendations

Based on these findings, we recommend giving students the option to get hints when they are testing themselves. It will make them choose testing more often, which should increase their learning, and it will also make learning more fun, which might increase their motivation to study. We envision instructors making more use of hints in worksheets, questions at the end of textbook chapters, flashcards, and a variety of digital study aides that resemble Quizlet. The students themselves might also benefit by finding ways to give themselves hints as they test themselves.

These recommendations need to be qualified in more than one way, however. First, hints are probably especially important when the participants would otherwise fail to answer most of the test questions without hints. Thus, the hints might be most useful when the learners are just starting to learn the material, or the material is very difficult. When a learner can get the answers right without hints, they will probably choose test trials with or without hints, so the hints might not hurt, but they might not help either.

On a related note, making hints too easy in the wrong way is another danger. In Experiment 4, it was possible to guess the answer based on the hint, even if one did not remember having studied the word pairs previously. In this case, participants tended to learn less from hint trials than test trials. In short, hints that make the answer guessable (e.g., king-q__en) could potentially impair learning (compared to test trials) and may need to be avoided. For this reason, further research is needed to verify that hints have the same effects with authentic educational materials as they do with word pairs.

Another important issue is that of the retention interval. In the present experiments, final test performance was assessed after a brief delay (2 min). Research has shown that testing is particularly effective across longer retention intervals, at least when feedback is not given (see Toppino & Cohen, 2009). However, this finding is not relevant here because feedback was given in the present study. When feedback is given, the benefits of tests (compared to restudy) do not increase as the retention interval increases (Kornell, Bjork, & Garcia, 2011). Thus, it is likely that the effects shown in this paper would have been similar with longer retention intervals, but only further research would confirm this.

Finally, we gave our participants the option to use hints, but we did not force this option on them. We do not know whether this affected the study since we never tried forcing the use of hints, but we suspect that participants will enjoy learning more when they get to choose how they want to study. Furthermore, Tullis, Fiechter, and Benjamin (2018) showed that tests are more effective than presentation trials overall, but forcing them to take a test does not enhance learning when participants do not want to be tested.

### A tale of two approaches to behavior modification

Here, we will highlight differences between two ways of getting students to test themselves, that is telling students that testing is a valuable way to learn, which we chose not to do, versus making students want to test themselves, as we did here. A great deal of ink has been spilled in the campaign to educate students and teachers about the benefits of tests. There are high-profile research articles (e.g., Dunlosky, Rawson, Marsh, Nathan, & Willingham, 2013; Pashler et al., 2007), newspaper articles (e.g., Carey, 2010), and books (Boser, 2017; Brown, Roediger, & McDaniel, 2014; Carey, 2015; Rhodes, Cleary, & Delosh, 2020). These are all undoubtedly good things. One big advantage of this approach—that is, spreading the word about the benefits of retrieval—is reach. Books and articles can reach a lot of people. Students can then, hopefully, use their newly found knowledge to make better use of their own study time, by testing themselves and so forth.

However, making students want to test themselves, rather than telling them that they should test themselves, has two advantages. First, a student’s beliefs do not always match their decisions; sometimes students choose to study in ways that they do not think are the most efficient (Kornell & Son, 2009). In other words, changing a student’s beliefs about the benefits of testing might not change how they chose to study.

There is a related problem with telling people that testing is good for them. We argue that students already think testing is good for them. We hypothesize that the reason they chose restudy, instead of testing, is not because they think testing is bad. Rather, it is because they are trying to avoid failure.

Although we did not directly measure whether avoidance of failure is what caused our participants to use hints, our data are consistent with this interpretation. First, self-testing was only the popular choice when hints were available (i.e., when the likelihood of getting the answer correct increased). Second, we analyzed initial test performance across experiments. If participants wanted to avoid failure, and chose to test themselves on items on which they expected to get the answer right, then they should show higher initial test performance when they were allowed to choose which items to test themselves on compared to when they were forced to self-test (as they were in Experiments 3a, 3b, and 4). The data support this hypothesis. For instance, in Experiment 2 (self-test by choice), initial test performance in the zero-letter condition was around 24% but it was only 12% and 10% in Experiments 3a and 3b, respectively. Additionally, two-letter performance in Experiment 1 was approximately 48% compared to 23% and 16% in Experiments 3a and 3b, respectively. These numbers are consistent with the notion that learners choose to test themselves when the odds of being correct are higher, although this should be taken lightly since they rely on cross-experimental comparisons, which are not ideal.

Future research can investigate situational factors that affect test avoidance. For example, people might be affected by the stakes of the situation. Someone who avoids using self-testing when the stakes are high (e.g., a medical student treating a patient) might be comfortable failing during a low-stakes quiz (e.g., a medical student at a pub quiz). Researchers have argued that medical students should employ retrieval practice as a study strategy (Larsen, Butler, & Roediger III, 2008), but these students may be reluctant to do so due to fear of failure. It has been shown that low-stakes quizzing can reduce test anxiety (Agarwal, D’Antonio, Roediger III, McDermott, & McDaniel, 2014), but that is irrelevant if students avoid self-testing altogether. Interestingly, testing with hints may reduce test anxiety by alleviating the fear of failure associated with testing and could be a useful option for those suffering from test anxiety. In summary, avoidance of failure does seem to be able to explain why students have been shown to prefer restudy in some situations.

If failure is what students are trying to avoid, then the best way to make them do more self-testing might be to convince them that they should embrace failure. Telling students something they already know—that they should test themselves sometimes—will not have much impact on learning.

Second, and more important, telling people what is best for them does not necessarily change their behavior for very long. Saying you should test yourself without making it fun is like saying you should eat your spinach without making it taste good. For a student, it will probably mean self-testing requires willpower and self-control. Because it is difficult to maintain self-control in the long term (e.g., Hoch & Loewenstein, 1991; Mischel, Shoda, & Rodriguez, 1989), we tried to remove self-control from the equation. We hoped our learners would test themselves not because it was the right thing to do, but because they wanted to. In other words, we tried to use hints to make the spinach taste good.

### Theoretical implications

We think these results have three sets of theoretical implications: people like to be tested; any test trial that meets two simple criteria will be equally effective; and retrieval effort might not affect learning. We will discuss each of these in turn.

First, it is often claimed that, when people study, they prefer restudy over self-testing (Geller, et al., 2018; Hartwig & Dunlosky, 2012; Karpicke, et al., 2009; Karpicke & Roediger, 2008; Kornell & Bjork, 2007; Kornell & Son, 2009; Morehead et al., 2016; Wissman et al., 2012). We think this idea has been painted with too broad a brush. General statements about whether people appreciate the value of testing, or want to be tested, or choose testing, are bound to be inaccurate because people’s preferences for restudy versus testing depend on the circumstances. For example, one must surely consider the kind of material being studied (e.g., people probably like to test themselves more when studying vocabulary than when reading a novel), but that is beyond the scope of this article. The circumstance we focused on was the person’s chance of getting the answer correct.

When we looked more specifically, our data suggested that, at least with simple word pairs, testing was more popular than restudy. That is, most people’s favorite option was to take a test that they could get right; this option was more popular than a test they could not get right or a restudy trial. Consistent with previous research, restudy trials were more popular than tests that the participants could not get right, but this comparison leaves out the participants’ favorite option. Therefore, we disagree with the idea that people underestimate the value of testing, or avoid testing themselves when they study. They do dislike and avoid something, but it is being wrong, not taking tests.

The second main theoretical implication of our results has to do with the finding from Experiments 3a and 3b that hints did not diminish the benefits of retrieval. This finding fits with Kornell and Vaughn’s (2016) two-stage model of learning from retrieval. The stages in this model are a legitimate retrieval attempt (stage 1) followed by exposure to the correct answer (stage 2). The model predicts that the full benefit of retrieval will be obtained any time these two conditions are met. In other words, if retrieval would provide a 10 percentage point boost in learning for a given word pair at a given time, then those 10 points will be obtained if there is a legitimate retrieval attempt followed by a chance to fully process the correct answer, regardless of other factors that might be at play. Previous research has supported this claim. One study showed that whether the retrieval attempt was successful or not did not affect learning (Kornell, Klein, & Rawson, 2015). Another showed that the amount of time one spends trying to retrieve an answer (i.e., the duration of stage 1) did not affect learning (Vaughn, Hausman, & Kornell, 2017).

Experiments 3 and 4 provided crucial additional support for this model by showing that hints, as long as they are not guessable, also did not affect learning. In Experiment 3, both stage 1 and stage 2 occurred in all of the retrieval conditions and, as predicted, the full benefit of retrieval was obtained regardless of whether there was no hint, a two-letter hint, or a four-letter hint. In Experiment 4, a legitimate retrieval attempt was not required, because participants could guess the answer based on semantic knowledge, so stage 1 did not always occur under the hint conditions. As predicted, the full benefit of retrieval was not obtained under the hint conditions. In short, Experiments 3 and 4 support the two-stage model by adding to the list of factors (retrieval success, retrieval duration, and now retrieval difficulty) that do not affect how much one learns from retrieval.

The third theoretical implication of these results has to do with the retrieval effort hypothesis (Pyc & Rawson, 2009; also see Bjork & Allen, 1970). According to this hypothesis, retrieval effort leads to learning, such that a difficult, high-effort retrieval produces more learning than does a relatively easy, low-effort retrieval (assuming the retrieval attempt is successful). A study by Pyc and Rawson (2009) supported this hypothesis. Their participants were to learn Swahili-English word pairs with either short practice lags (e.g., six intervening items) or long practice lags (e.g., 34 intervening items). Longer practice lags increased the amount of effort required during the retrieval attempts in the study phase. As predicted by the retrieval effort hypothesis, on the final test participants did better under the condition with longer practice lags.

Although Pyc and Rawson’s (2009) data are consistent with the retrieval effort hypothesis, there is an alternative explanation of their data. They showed that participants learned more from longer lags than shorter lags. They explain this finding based on retrieval effort, but it can also be seen as a spacing (or “lag”) effect, and a great deal of research has shown that longer lags lead to more learning than shorter lags (for a review, see Cepeda, Pashler, Vul, Wixted, & Rohrer, 2006). Moreover, lag effects can be explained by factors other than retrieval effort. For example, perhaps the benefit of the longer lags in Pyc and Rawson’s data came from a difference in accessibility (which was lower in the longer lag condition), not from a difference in retrieval effort (Bjork & Bjork, 1992). In other words, retrieval effort was correlated with learning, but it might not have caused learning. There is a third variable in this correlation, memory accessibility, that is known to influence learning. Accessibility and retrieval effort were confounded. Therefore, it is possible that retrieval effort did not affect learning directly, but only appeared as though it did, while what actually happened was that low accessibility under the long lag condition caused both retrieval effort to be high and learning to be high.

To overcome this third variable problem, and truly test whether retrieval effort has a causal impact on learning, accessibility needs to be held constant while retrieval effort is manipulated. The methodology of Experiments 3 and 4 achieved this; hints made retrieval less difficult and less effortful, but they did not affect accessibility. If retrieval effort has a causal effect on learning, then hints should have affected the amount participants learned. No such effect materialized in Experiments 3a or 3b. (This effect did occur in Experiment 4 but, as we have already explained, the effects in Experiment 4 can be explained based on guessability.)

In short, Experiments 3a and 3b may be the strongest test yet of the retrieval effort hypothesis, but this hypothesis was not supported. Thus, perhaps an amended version of the retrieval effort hypothesis is in order—retrieval effort can be positively correlated with learning, but retrieval effort per se might not cause learning. Further research is needed to look at these possibilities.

## Conclusion

One of the key ways to help students learn more is to get them to adopt better study habits. New habits that are unpleasant rarely last very long. The easiest habits to adopt are the ones that you enjoy. The research presented here suggests that giving people the opportunity to take hints when they test themselves can make them more likely to test themselves and more likely to be able to study in a way that they prefer. Teachers, app designers, students, and anyone who wants to make testing both fun and effective might do well to build hints—but not guessable hints—into their tests.

## Data Availability

All data and materials are available upon request.

## Appendix 1

**Table 2:** Cue-target pairs used in Experiments 1, 2, and 3

Cue	CDcount1	CDcount2	CDcount3	CDcount4	Target
area	handed	hatred	hanged	hacked	harmed
army	hooker	holler	hotter	hoover	holder
back	harder	hammer	harper	hamper	hanger
bath	talked	tasted	tapped	tagged	tailed
beer	shakes	shares	shines	shades	shapes
bird	better	beaver	bender	beeper	bearer
cell	member	merger	meaner	mercer	meager
city	sheets	shorts	shirts	shoots	shifts
data	denied	deemed	defied	dented	decked
debt	sticks	stinks	stocks	steaks	stacks
desk	grades	graves	grapes	graces	groves
dirt	dinner	diaper	differ	digger	dipper
disk	career	cancer	carter	caller	camper
exam	called	caused	canned	carved	cashed
fact	blades	blames	blazes	blokes	blares
film	killer	kisser	kinder	kicker	kidder
fish	states	stores	stones	stakes	stages
food	butter	burger	buster	bummer	butler
form	branch	breach	brunch	brooch	breech
game	powder	potter	poster	porter	poorer
gate	corner	cooler	cooper	copper	colder
gene	booked	bombed	boiled	bolted	bonded
girl	intact	insect	inject	infect	indict
goal	stayed	stoned	stated	stored	stared
hair	lawyer	larger	ladder	latter	lather
hall	proves	prices	prizes	prunes	probes
heat	ruined	rushed	rubbed	rugged	rusted
idea	server	seller	sexier	sender	seeker
king	chores	chimes	chases	chutes	chokes
lady	doomed	dotted	docked	dodged	downed
lake	heater	helper	healer	heller	heifer
life	seemed	served	sealed	seated	sensed
loss	proved	prayed	primed	prized	priced
love	banker	barber	banner	batter	banter
mall	backed	banged	bailed	banned	bagged
math	raised	rained	ragged	raided	ratted
meal	pulled	pushed	pumped	puffed	purged
meat	bother	border	bomber	boiler	booger
menu	horses	houses	hooves	homies	hordes
mode	damned	danced	darned	dawned	dashed
mood	solved	soaked	sorted	soiled	socked
news	passed	packed	parked	parted	padded
oven	dealer	deeper	dexter	deader	decker
part	wanted	walked	waited	wasted	warned
poem	forced	fooled	formed	forged	folded
poet	looked	locked	loaded	loaned	lodged
road	common	cotton	coupon	cocoon	cordon
role	matter	master	manner	marker	madder
song	robbed	rolled	rocked	rooted	routed
soup	danger	dancer	darker	dagger	damper
tale	winner	winter	wiener	wilder	wither
time	jammed	jacked	jailed	jazzed	jagged
town	scares	scores	scenes	scales	scones
unit	sister	silver	singer	sinner	simmer
user	buried	burned	busted	bumped	bugged
week	summer	suffer	supper	sucker	surfer
wife	cooked	copied	cooped	conned	cooled
wood	marked	mashed	mailed	masked	mapped
work	taller	talker	tanker	tanner	tamper
year	wicked	wished	winged	winked	willed

Note: The cue word is listed in the left-most column. The remaining columns list the five most common words (based on the numerical CDcount value within Subtlexus) for that letter combination (e.g., ha__ed). The final column is the fifth most popular word and represents the target used in the experiment

## Appendix 2

**Table 3:** Cue-target pairs used in Experiment 4

CUE	TARGET
bald	smooth
bill	dollar
bird	parrot
boil	bubble
book	school
butt	bottom
cage	prison
calm	serene
case	lawyer
crew	people
cure	remedy
dare	double
deer	forest
diet	skinny
disc	record
dope	stupid
drag	boring
dust	sneeze
face	pretty
fact	figure
file	folder
five	number
flow	stream
grow	shrink
heal	better
herd	animal
hill	valley
holy	priest
kick	soccer
kind	gentle
line	circle
lint	pocket
meet	gather
mint	flavor
move	travel
peak	climax
pest	rodent
pine	needle
plan	action
puff	powder
roam	around
rose	beauty
snap	button
soft	cuddle
span	length
spin	rotate
stem	branch
tape	scotch
tell	secret
tiny	little
tool	wrench
tour	france
type	letter
view	window
vote	ballot
walk	stroll
wall	street
whip	punish
wine	dinner
yell	holler

## Footnotes

1 Nebraska, Nevada, New Hampshire, New Jersey, New Mexico, New York, North Carolina, and North Dakota.

2 Maine, Maryland, Massachusetts, Michigan, Minnesota, Mississippi, Missouri, and Montana.

3 Alfred.

4 Belatrix Lestrange.

5 Mjolnir.

6 Dave Grohl.

7 Parr, Corleone, von Trapp.

8 Beck, Pearljam, Kelly Clarkson, Elvis Presley, Van Morrison, Prince, Donovan, Booker T. & the M.G.’s, Nick Drake, Led Zeppelin, The Beatles, AC/DC, Amy Winehouse, The Rolling Stones, Metallica, Soundgarden, Cream.
